# Combined versus sequential penetrating keratoplasty and cataract surgery for herpes simplex keratitis: a retrospective study

**DOI:** 10.3389/fmed.2023.1190485

**Published:** 2023-07-21

**Authors:** Yani Wang, Jun Cheng, Nannan Yang, Ting Li, Yanling Dong, Lixin Xie

**Affiliations:** ^1^Medical College, Qingdao University, Qingdao, China; ^2^Qingdao Eye Hospital of Shandong First Medical University, Qingdao, China; ^3^State Key Laboratory Cultivation Base, Shandong Provincial Key Laboratory of Ophthalmology, Eye Institute of Shandong First Medical University, Qingdao, China

**Keywords:** herpes simplex keratitis, cataract surgery, penetrating keratoplasty, graft survival, endothelial cell density

## Abstract

**Purpose:**

To compare the surgical outcomes of combined penetrating keratoplasty (PK) and cataract surgery with those of sequential surgery (cataract surgery after PK) for herpes simplex keratitis (HSK).

**Methods:**

The medical records of consecutive patients diagnosed with HSK who underwent combined or sequential PK and cataract surgery in active and stable stages between June 2015 and June 2022 were reviewed retrospectively. Complications, graft survival, endothelial cell density (ECD), and final BCVA were compared and analyzed between both surgical methods in each stage.

**Results:**

A total of 171 eyes of 171 patients were enrolled, including active stage (69 combined, 46 sequential) and stable stage (34 combined, 22 sequential). The average follow up was 24.2 ± 15.8 months (range, 3 months – 48 months). The final BCVA had obvious improvement and the postoperative ECD was not different in combined and sequential groups of each stage. In sequential group of active stage, 66.7% of persistent epithelial defects and 50% of HSK recurrence occurred within 3 months after cataract surgery; nevertheless, compared to that in sequential group, capsular rupture (*p* = 0.021), persistent epithelial defects (*p* = 0.027), and HSK recurrence (*p* = 0.035) occurred more frequently in combined group, leading to a lower graft survival rate (*p* = 0.045); at the last visit, 46.4 and 67.4% of grafts remained clear in combined and sequential groups, respectively. By contrary, 82.4 and 50.0% of grafts remained clear in stable stages of combined and sequential groups at the last visit, respectively, and a higher graft survival rate was observed in combined group (*p* = 0.030).

**Conclusion:**

Although the postoperative ECD is not different between two surgical groups in each stage, sequential surgery in active stage of HSK seems to have advantages in less complications and higher graft survival rate, whereas combined surgery in stable stage has a better outcome than that in sequential surgery.

## Introduction

Penetrating keratoplasty (PK) is an effective surgical approach for removing diseased cornea and improving symptoms. Corneal diseases requiring PK are usually accompanied with cataract. Especially, progressed or mature cataract with a risk of secondary glaucoma or uveitis requires surgical removal (1). Despite combined or sequential PK and cataract surgery is recognized as effective and well-established surgical treatment for corneal diseases accompanied with cataract, cataract surgery performed in patients with preexisting corneal diseases, still has a potential risk to leading to bad outcomes for visual acuity and increasing the risk of complications (2). Combined PK and extracapsular cataract extraction (ECCE) with intraocular lens (IOL) implantation, defined as a single surgical procedure, has been recognized to have remarkable advantages for faster visual rehabilitation (3, 4), whereas considering the more satisfactory effect in safety and visual improvement, ECCE combined with IOL insertion after PK is more preferred for clinicians (5). Therefore, whether to perform the combined procedure, or take a staged step for corneal diseases with cataract, still remains a debated topic.

Herpes simplex keratitis (HSK) is a leading cause of corneal blindness across the world (6, 7). Particularly in developing countries, the incidence of HSK has become much greater (8). HSK can evolve from epithelial keratitis to stromal keratitis, developing corneal perforation or corneal scarring, undoubtedly requiring PK to sustain visual acuity and eyeball integrity (9). Epithelial defects, recurrence of HSK, and graft rejection after PK are main risks for graft failure in patients with HSK (10). Cataract is a common complication in patients with HSK, and cataract surgery has been reported to be a risk for HSK recurrence and infection (11). However, limited information is available on the surgical outcomes of combined or sequential PK and ECCE+IOL for HSK. Thus, this study aimed to analyze and compare the surgical outcomes of combined PK and ECCE +IOL with those of sequential surgery (ECCE+IOL after PK) for HSK.

## Materials and methods

### Patients selection

The study was approved by the ethics committee of Qingdao Eye Hospital (2021–14) and was conducted in accordance with the principles of the Declaration of Helsinki. The medical records of patients with HSK who underwent combined or sequential PK and cataract surgery in the active and stable stages at Qingdao Eye Hospital between June 2015 and June 2022 were retrospectively reviewed. The exclusion criteria for combined surgery included: the patients who were diagnosed with HSK complicated with other corneal diseases that required PK, and patients diagnosed with HSK undergoing repeat PK. The exclusion criteria for sequential surgery included: patients with HSK undergoing sequential cataract surgery after PK because of secondary glaucoma.

### Pathological staging of HSK and surgical indications

1. Pathological staging of HSK: active phase presented as ciliary congestion, corneal inflammatory infiltration and edema, deep stromal neovascularization, corneal ulceration, or corneal perforation. Stable phase presented as no conjunctival congestion, mild corneal edema, and clear boundary of corneal scar.
2. Indications for PK: PK was performed for full-thickness corneal scars in stable stage to remove the diseased cornea and improve visual acuity. In active stage, PK was performed for corneal perforation or full-thickness corneal ulcerations after the inflammation was initially controlled.
3. Indications for cataract surgery: combined cataract surgery was performed in patients with progressed or mature cataract that was confirmed before and during the operation. Sequential cataract surgery was performed at least 3 months after PK, when the progression of cataract in patients with a transparent graft and no other complications [endothelial cell density (ECD) > 600 cells/mm^2^]obviously affected the visual acuity.

### Surgical technique and clinical care

Donor corneas were obtained from the Eye Bank of Qingdao Eye Hospital. Combined and sequential surgeries were performed by the two experienced surgeons. The surgical steps of PK combined ECCE with IOL implantation and sequential cataract surgery (ECCE combined with IOL implantation) after PK were consistent with our previous studies reported (12, 13).

Before surgery, acyclovir 0.25 g was administered intravenous drip three times a day in active stage. Acyclovir eye drops (Wujing®, Wujing Pharmaceutical Co., Wuhan, China) were used once every 2 h and ganciclovir ophthalmic gel (Likeming®, Keyi Pharmaceutical Co., Hubei, China) was used once time before bedtime. 20% mannitol 250 mL was administered intravenous drip to sufficiently reduce the intraocular pressure.

After PK, intravenous administration of 1.5 g cefuroxime sodium was administered twice a day and 150 mg hydrocortisone was administered once a day lasting for 3 days. Oral acyclovir dosage of 400 mg was administered twice daily, lasting for 2 weeks. Topical antibiotics were given four times a day for 2 weeks after surgery. Steroids were administered topically for at least 2 years after PK. Acyclovir eye drops were used twice a day or ganciclovir ophthalmic gel was used once time before bedtime, lasting for at least 1 year. At 1 week postoperatively, all patients were administered 1% cyclosporine A eye drops (Tiankeming®, North China Pharmaceutical Co., Shijiazhuang, China) or 0.1% tacrolimus eye drops (TALYMUS®, Senju, Osaka, Japan) four times a day, lasting for at least 2 years.

### Main outcome evaluation

Pre- and post-operative intraocular pressure (IOP), ECD (postoperative 3, 6, 12, 24, 36, and 48 months), intra-and post-operative complications, graft survival rate, and final best corrected visual acuity (BCVA) were recorded. Corneal epithelial integrity was evaluated with fluorescein staining under slit-lamp microscopy. IOP were measured using non-contact tonometer (TX-20, Canon Tokyo, Japan). The endothelium was imaged using a noncontact specular microscope (SP-3000P, Topcon Tokyo, Japan), and approximately 60 cells were analyzed for the mean cell density. Graft failure was defined as irreversible loss clarity of central graft, regardless of the level of visual acuity (14). Visual acuity was converted into logarithm of the minimum angle of resolution (Log Mar) units for statistical analysis.

### Statistical analysis

Data are expressed as percentages and mean ± SD. SPSS software (version 24.0; SPSS, Inc. Chicago, IL, United States) was used for the statistical analyses. Preoperative and intraoperative characteristics, and postoperative complications were analyzed with the Chi-square test, Fisher’s exact test or chi-square continuity correction. Independent-sample *t*-test was used for the comparison of IOP between two surgical groups of each stage on condition of normal distribution. Manan–Whitney *U* test was used for analysis of ECD at each follow-up time point and mean graft survival time. The Kaplan–Meier curve method and Mantel-Cox test were used to assess the cumulative graft survival rate. Statistical significance was set at *p* < 0.05.

## Results

### Patient demographics

A total of 171 patients (171 eyes) with HSK undergoing combined or sequential PK and cataract surgery were enrolled in this study including active stage (69 combined, 46 sequential) and stable stage (34 combined, 22 sequential). The ratio of male and female was 1.8:1, with an average age of 61.6 ± 9.3 years (range, 43–74 years). The average follow up was 24.2 ± 15.8 months (range, 3 months – 48 months). The demographics and intraoperative clinical characteristics of patients are shown in Table 1.

**Table 1:** Preoperative and intraoperative clinical characteristics of patients between both surgical methods for HSK.

	Active stage		*p* value	Stable stage		*p* value
	Combined group (*n* = 69)	Sequential group (*n* = 46)		Combined group (*n* = 34)	Sequential group (*n* = 22)	
Sex			0.340			0.625
Male	42 (60.7%)	32 (69.6%)		21 (61.8%)	15 (68.2%)	
Female	27 (39.3%)	14 (30.4%)		13 (38.2%)	7 (31.8%)	
Age, mean ± SD, range, year	62.3 ± 8.8 (47–76)	66.1 ± 6.3 (52–74)	0.300	63.5 ± 9.9 (51–72)	58.8 ± 8.8 (49–70)	0.360
Preoperative BCVA (log Mar)			0.165			0.586
4	4 (5.8%)	0 (0%)		0 (0%)	0 (0%)	
3	29 (42.0%)	16 (34.8%)		11 (32.4%)	5 (22.7%)	
2.1–2.9	31 (44.9%)	21 (45.6%)		18 (52.9%)	15 (68.2%)	
1.3–2	4 (5.8%)	6 (13.0%)		3 (8.8%)	2 (9.1%)	
1–1.2	1 (1.4%)	3 (6.5%)		2 (5.9%)	0 (0%)	
Preoperative IOP(mmHg)	17.6 ± 4.5 (10–25)	18.2 ± 5.9 (11–28)	0.733	16.6 ± 3.8 (11–22)	14.6 ± 2.9 (10–21)	0.061
**Intraoperative characteristics**						
Graft diameter ≥ 9 mm	16 (23.2%)	9 (19.6%)	0.644	5 (14.7%)	4 (18.2%)	1.000
IOL implantation	41 (59.4%)	37 (80.4%)	0.018	32 (94.1%)	17 (77.3%)	0.063
Capsular rupture	24 (34.8%)	7 (15.2%)	0.021	1 (2.9%)	5 (22.7%)	0.030
Vitreous loss	19 (27.5%)	7 (15.2%)	0.122	1 (2.9%)	4 (18.2%)	0.072

BCVA, best corrected visual acuity; IOL, intraocular lens; IOP, intraocular pressure.

There were no significant differences in age, sex, preoperative IOP, or preoperative BCVA between the two surgical groups in both stages. However, a lower implantation rate of IOL with higher prevalence of capsular rupture was observed in combined group of active stage (Table 1).

### Visual outcome

**Figure 1:**
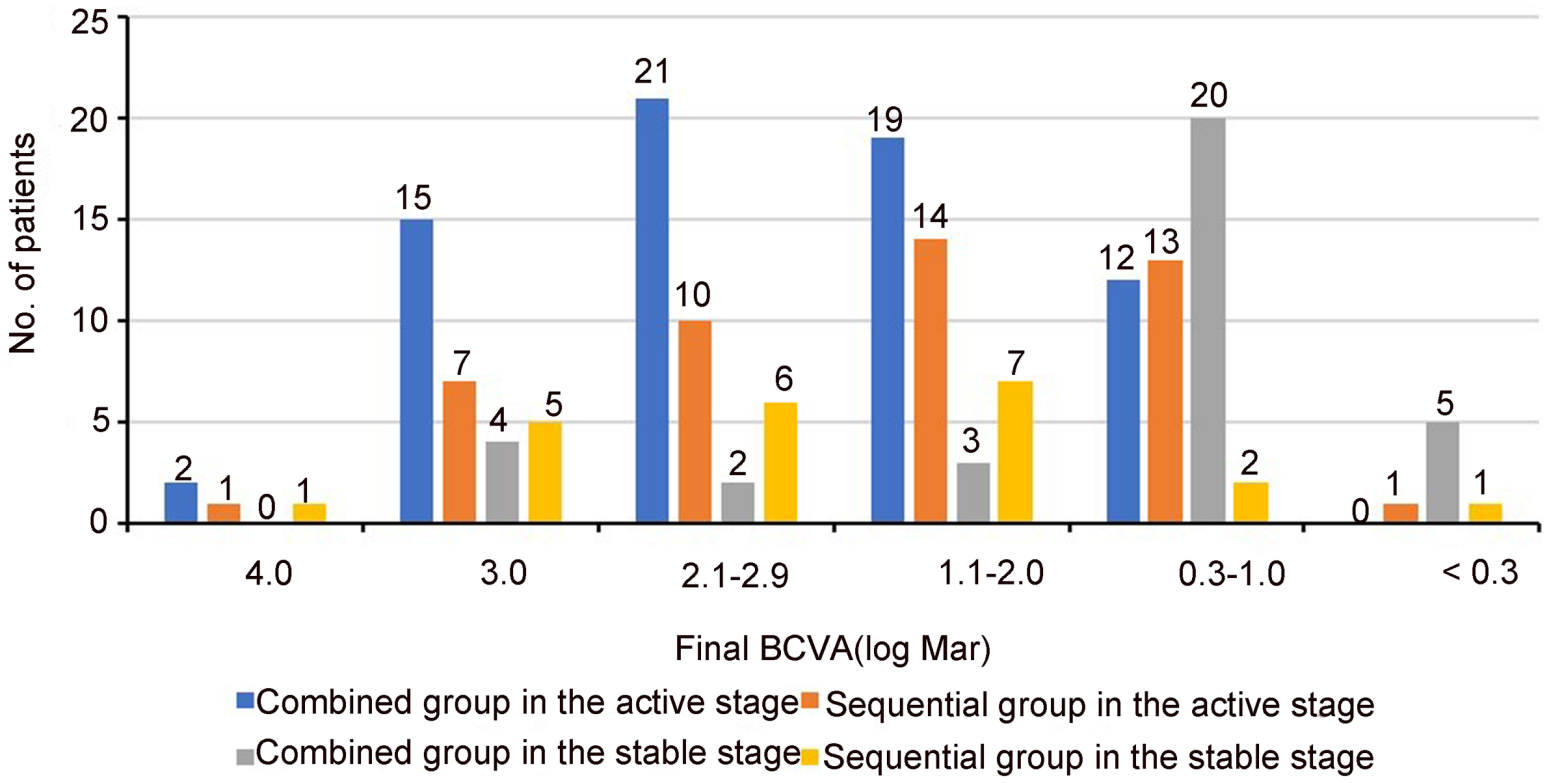
Graph showing the distribution of BCVA(log Mar) at final follow-up in two stages of HSK.

The visual outcomes are summarized in Table 2; Figure 1. The mean BCVA (log Mar) at the final follow-up had significant improvement than that before surgery in both stages (*p* < 0.05; Table 2). Nevertheless, final BCVA ≤1.0 (log Mar) in combined group of active stage were observed only in 12 eyes (17.4%), whereas 25 eyes (73.5%) with final BCVA ≤1.0 (log Mar) were observed in combined group of stable stage.

**Table 2:** Comparison of BCVA(log Mar) between the final follow-up and pre-surgery in both stages of HSK.

	Combined group		*p* value	Sequential group		*p* value
	Before surgery	Final follow-up		Before surgery	Final follow-up	
Active stage	2.9 ± 0.7 (log Mar)	1.7 ± 0.8 (log Mar)	<0.001	2.1 ± 0.8 (log Mar)	1.2 ± 0.7 (log Mar)	0.027
Stable stage	2.3 ± 0.7 (log Mar)	0.7 ± 0.3 (log Mar)	<0.001	2.3 ± 0.9 (log Mar)	1.3 ± 0.9 (log Mar)	0.017

### Endothelial cell density

**Figure 2:**
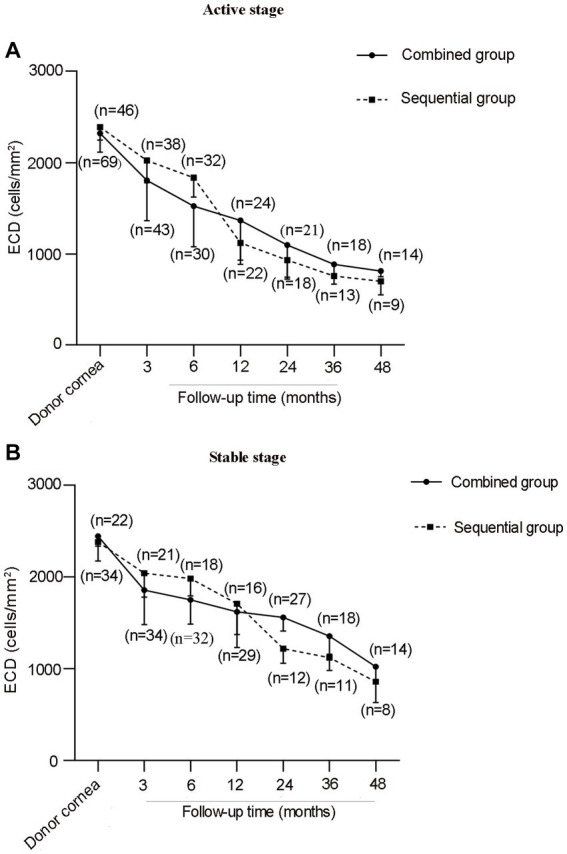
Graph showing the change of ECD after two surgical groups in active stage **(A)** and stable stage **(B)** of HSK.

The mean interval time of cataract surgery after PK in sequential groups of active and stable stages was 9.3 ± 7.5 months (range, 3–18 months) and 12.1 ± 4.0 months (range, 6–18 months), respectively. At the final follow-up, the average IOP in combined group of stable stage was 18.1 ± 5.2 mmHg (range, 10–22 mmHg), compared with 15.9 ± 4.0 mmHg in sequential group (range, 12–21 mmHg; *p* = 0.132); the mean IOP in combined group of active stage was 15.4 ± 4.0 mmHg (range,11–21 mmHg), compared with 16.7 ± 2.6 mmHg in sequential group (range,10–20 mmHg; *p* = 0.344). Under this condition, we compared the ECD between both surgical groups of each stage. Interestingly, although the mean ECD in sequential groups of both stages was higher before cataract surgery at each follow-up time point and obviously decreased after cataract surgery, there was no statistical difference in ECD at pre-surgery, postoperative 1, 3, 6, 12, 24, 36, and 48 months between combined and sequential groups of active stage (*p* = 0.383, 0.180, 0.197, 0.294, 0.237, 0.216, 0.225) and stable stage (*p* = 0.355, 0.174, 0.166, 0.412, 0.082, 0.167, 0.155; Figure 2).

### Complications

Postoperative complications are presented in Table 3. In active stage, HSK recurrence (28 eyes, 40.6%) was the most common complication in combined group, followed by persistent epithelial defects (21 eyes, 30.4%) and graft ulcerations (16 eyes, 24.6%); while HSK recurrence (10 eyes, 21.7%) was the most common complications in sequential group, followed by persistent epithelial defects (9 eyes, 19.6%) and endothelial decompensation (9 eyes, 19.6%). Moreover, 41% (16/39) of postoperative complications in sequential group occurred within 3 months after cataract surgery, including persistent epithelial defects (66.7%, 6/9), HSK recurrence (50%, 5/10), graft rejection (42.9%, 3/7), endothelial decompensation (11.1%, 1/9), and graft ulcerations (11.1%, 1/9).

**Table 3:** Distribution and comparison of postoperative complications between both surgical methods.

Postoperative complications	Active stage		*p* value	Stable stage		*p* value
	Combined group (*n* = 69)	Sequential group (*n* = 46)		Combined group (*n* = 34)	Sequential group (*n* = 22)	
Graft rejection	8 (11.6%)	7 (15.2%)	0.572	3 (8.8%)	3 (13.6%)	0.161
Persistent epithelial defects	21 (30.4%)	9 (19.6%)	0.027	1 (2.9%)	5 (22.7%)	0.030
Graft ulceration	17 (24.6%)	8 (17.4%)	0.356	2 (5.9%)	1 (4.5%)	1.000
Endothelial decompensation	7 (10.4%)	9 (19.6%)	0.183	1 (2.9%)	4 (18.2%)	0.072
Recurrence of HSK	28 (40.6%)	10 (21.7%)	0.035	9 (26.5%)	7 (31.8%)	0.665

In stable stage, the most common complications in combined and sequential groups were HSK recurrence in 9 eyes (26.5%) and 7 eyes (31.8%), respectively, with no significant difference between two surgical groups (*p* = 0.665). However, persistent epithelial defects were observed in 5 eyes (22.7%) in sequential group, compared with 1 eye (2.9%) in combined group (*p* = 0.030); moreover, 27.8% (5/18) of postoperative complications occurred within 3 months in sequential group after cataract surgery, including persistent epithelial defects (60.0%, 3/5), HSK recurrence (40%, 2/5), and graft rejection (33.3%, 1/3).

### Graft survival

**Figure 3:**
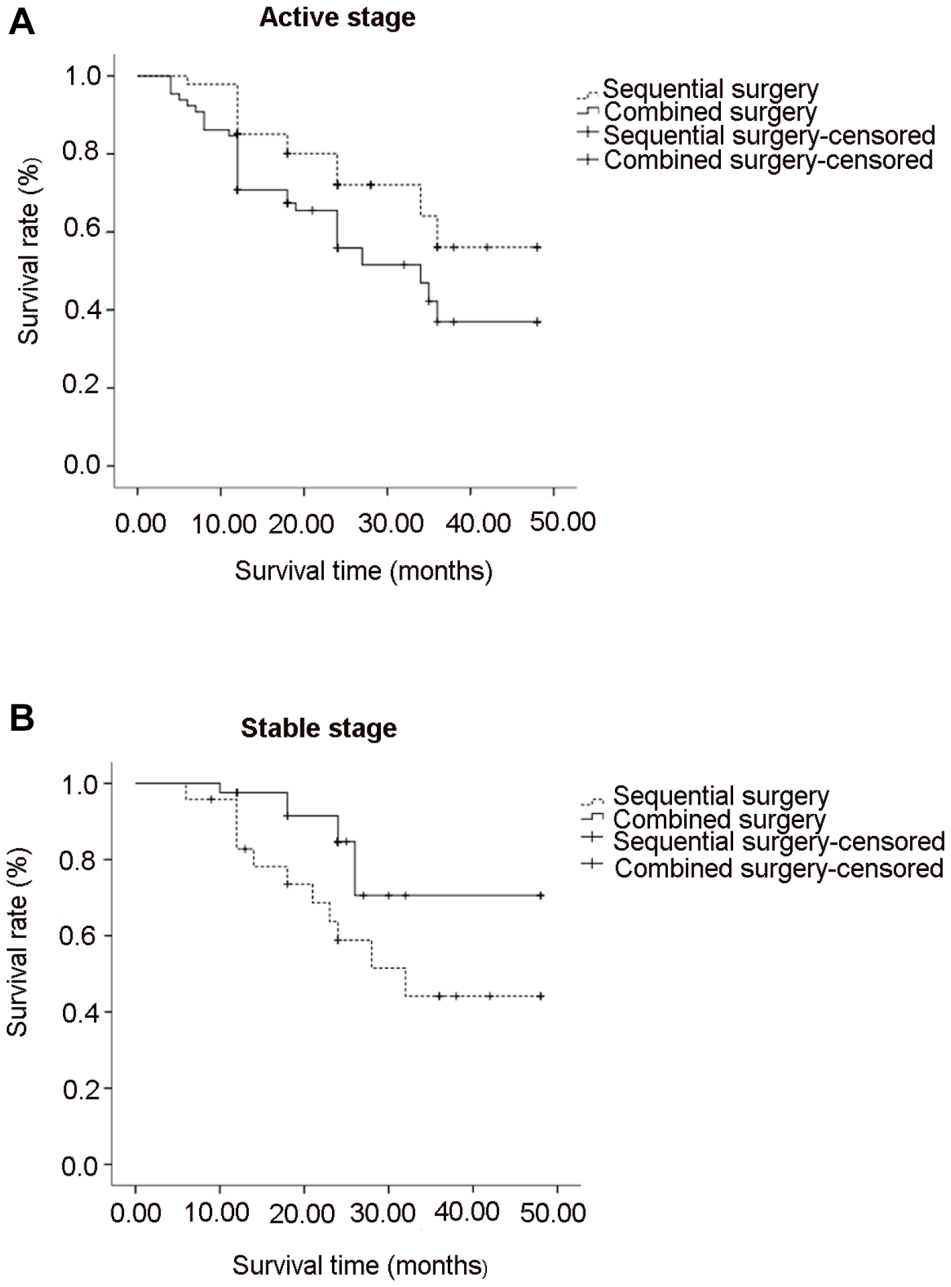
Graph showing graft survival rate after combined and sequential surgeries in active stage **(A)** and stable stage **(B)** of HSK.

The mean survival time of grafts in cases with postoperative complications is shown in Table 4. At the last visit of active stage, graft remained clear in 32 eyes (46.4%) and 31 eyes (67.4%) in combined and sequential groups, respectively, with a significant difference in cumulate graft survival rate (log-rank, 4.006; *p* = 0.045; Figure 3A). The graft survival rate at 2 years was 67.4 and 72.1% in the two surgical groups, respectively. The main reasons for graft failure in combined group included HSK recurrence (*n* = 15), persistent epithelial defects (*n* = 9), endothelial decompensation (*n* = 7), and graft ulcerations (*n* = 6); the main causes for graft failure in sequential group included endothelial decompensation (*n* = 9), persistent epithelial defects (*n* = 4), and recurrence of HSK (*n* = 2).

**Table 4:** Comparison of mean graft survival time in cases with postoperative complications after both surgical methods.

Postoperative complications	Mean graft survival time in active stage (months)		*p* value	Mean graft survival time in stable stage (months)		*p* value
	Combined group (*n* = 69)	Sequential group (*n* = 46)		Combined group (*n* = 34)	Sequential group (*n* = 22)	
Graft rejection	23.0 ± 9.2	22.0 ± 7.0	0.832	36.0 ± 11.1	21.8 ± 8.7	0.113
Persistent epithelial defects	16.1 ± 5.8	31.0 ± 10.5	0.008	28.0	15.7 ± 5.1	–
Graft ulceration	22.1 ± 10.6	27.3 ± 12.7	0.518	18.5 ± 3.5	26.0	–
Endothelial decompensation	26.2 ± 11.1	22.0 ± 8.9	0.465	34.0	16.2 ± 2.5	–
Recurrence of HSK	15.3 ± 8.3	31.6 ± 7.6	0.002	24.2 ± 5.6	19.3 ± 10.7	0.577

In stable stage, graft remained clear in 28 eyes (82.4%) and 11 eyes (50.0%) in combined and sequential groups, respectively, with a significant difference in cumulate graft survival rate (log-rank, 4.699; *p* = 0.030; Figure 3B). The graft survival rate at 2 years was 84.7 and 57.3% in combined and sequential groups, respectively. Graft ulcerations (*n* = 2), recurrence of HSK (*n* = 3), and endothelial decompensation (*n* = 1) were the main causes for graft failure in combined group; Endothelial decompensation (*n* = 4), persistent epithelial defects (*n* = 4), and HSK recurrence (*n* = 3) were the main causes for graft failure in sequential group.

## Discussion

HSK is a common, potentially blinding corneal diseases characterized by recurrent infection (9). Recurrent HSK can lead to stromal inflammation or neurotrophic keratitis, resulting in corneal perforation or full-thickness corneal scarring that requires PK (15). Combined or sequential PK and cataract surgery are well-established surgical treatments for corneal diseases coexisting with cataract due to their own advantages. Nevertheless, given the clinical characteristics of HSK in acute and stable stages, whether there are differences in surgical outcomes, including complications, graft survival, and corneal endothelial cell density, between combined and staged surgeries in acute and stable stages of HSK have not been previously reported to our knowledge.

In view of the characteristics of HSK, especially in the active inflammatory stage, the reasonable use of antiviral drugs and glucocorticoids before the operation contribute to help alleviate the ocular inflammation. During the operation, the maintenance of infraorbital pressure and intraoperative appropriate use of viscoelastic agents are important for avoiding too much stimulation of iris and forming anterior chamber to create favorable conditions for cataract surgery. Despite all this, cataract surgery performed in patients with a history of HSK still has a great challenge (11). Complicated operation environment, such as iris dystrophy and posterior synechiae formation of iris, obviously increases the risk of capsule rupture during operation. As observed in our series, the rupture of posterior capsule during the operation occurred more frequently in active inflammatory stage.

In the present study, graft survival rate was observed in 84.7% of cases at 2 years in combined surgery group of stable stage, which was similar to previous reports in the literature (16). A higher graft survival rate is partly attributed to a healthy environment of ocular surface without other complications. The inflammatory status of the eye during operation may execrate the risk for postoperative complications, including epithelial defects and graft rejection (17). Furthermore, cataract surgery has been reported to increase the risk for corneal complications, such as corneal epithelial lesions (18) and HSK recurrence (19). In our series, compared to that in sequential group of active stage, a higher prevalence of persistent epithelial defects and recurrence of HSK with a higher graft failure rate occurred after combined surgery. Moreover, we found that a high percentage of persistent epithelial defects and HSK recurrence also occurred within 3 months after cataract surgery in sequential groups of both stages. These results indicate that cataract surgery may contribute to postoperative complications after PK in patients with HSK, especially in active stage.

In our series, HSK recurrence rate ranged from 21.7 to 40.6% after surgery, which was consistent with the range from 19 to 61% reported in the literature (20, 21). Cho et al. (22) have reported that the corneal nerves interrupted due to surgical access from temporal incision for cataract surgery may be one of main risks for triggering reactivation of HSK. Additionally, frequent recurrences and time quiescent prior to surgery were thought to be main risk factors for HSK recurrence (11, 23). In our observations, the mean intervals between PK and cataract surgery in active and stable stages were 9.3 and 12.1 months, respectively, and we found that the prevalence of HSK recurrence occurring within 3 months after cataract surgery in sequential groups of active and stable stages was 45.5 and 20%. These results suggest that cataract surgery performed for HSK, especially in active stage, may have a higher risk for HSK recurrence. Therefore, perioperative treatment and timely intervention postoperatively may be important strategies to decrease HSK recurrence.

Cataract surgery is undoubtedly one important risk factor for endothelial injury. Endothelial cell decompensation is a frequently observed postoperative complication for late graft failure (24). Endothelial cell loss rate for non-inflammatory corneal diseases reported in the literature is varied after PK, ranging from 20.3 to 22.5% at 6 months (25–27), reaches the highest loss rate approximately at 40% in the first year, and then decreases gradually during the subsequent follow-up (28, 29). However, other studies have reported that the average corneal endothelial cell loss rate can reach 75% at 5 years after PK (30). As a single procedure, combined surgery is thought to can avoid secondary damage to the donor endothelium during the operation. However, the effects of combined or sequential surgery on the endothelial cell loss is still controversial. In our series, although the ECD decreased obviously after cataract surgery in sequential group, ECD was not different at overall time points between the two surgical treatments in both stages. These results indicate that combined or sequential surgery has no different effects on the endothelial cell loss in patients with HSK. However, sequential surgery performed in patients with low ECD may have a risk of continued endothelial cells loss, thus leading to endothelial decompensation.

In summary, we conclude that cataract surgery performed after PK in HSK may increase the risk of postoperative complications. Combined surgery, as a single surgery, seems to have advantages in less complications and higher graft survival rate in stable stage of HSK, whereas sequential surgery in active stage of HSK seems to have a higher graft survival compared to that after combined surgery.

## Data availability statement

The original contributions presented in the study are included in the article/supplementary material, further inquiries can be directed to the corresponding authors.

## Ethics statement

The studies involving human participants were reviewed and approved by The ethics committee of Qingdao Eye Hospital. Written informed consent for participation was not required for this study in accordance with the national legislation and the institutional requirements.

## Author contributions

YW analyzed the data and took responsibility for the integrity and accuracy of the information, and drafted the manuscript. TL and NY participated in information gathering. JC, YD, and LX conceived and designed the study. JC and YD revised the manuscript. All authors contributed to the article and approved the submitted version.

## Funding

This study was supported by the Academic Promotion Plan of Shandong First Medical University (2019zl001).

## Conflict of interest

The authors declare that the research was conducted in the absence of any commercial or financial relationships that could be construed as a potential conflict of interest.

## Publisher’s note

All claims expressed in this article are solely those of the authors and do not necessarily represent those of their affiliated organizations, or those of the publisher, the editors and the reviewers. Any product that may be evaluated in this article, or claim that may be made by its manufacturer, is not guaranteed or endorsed by the publisher.
